# Spin-state reconfiguration induced by alternating magnetic field for efficient oxygen evolution reaction

**DOI:** 10.1038/s41467-021-25095-4

**Published:** 2021-08-10

**Authors:** Gang Zhou, Peifang Wang, Hao Li, Bin Hu, Yan Sun, Rong Huang, Lizhe Liu

**Affiliations:** 1 Key Laboratory of Integrated Regulation and Resources Development on Shallow Lakes, Ministry of Education, College of Environment, Hohai University, Nanjing, People’s Republic of China; 2 Department of Chemistry, University of Utah, Salt Lake City, UT USA; 3 Jiangsu Key Laboratory for Nanotechnology and Collaborative Innovation Center of Advanced Microstructures, National Laboratory of Solid State Microstructures, Nanjing University, Nanjing, People’s Republic of China

**Keywords:** Environmental sciences, Energy science and technology

## Abstract

Oxygen evolution reaction (OER) plays a determining role in electrochemical energy conversion devices, but challenges remain due to the lack of effective low-cost electrocatalysts and insufficient understanding about sluggish reaction kinetics. Distinguish from complex nano-structuring, this work focuses on the spin-related charge transfer and orbital interaction between catalysts and intermediates to accelerate catalytic reaction kinetics. Herein, we propose a simple magnetic-stimulation approach to rearrange spin electron occupation in noble-metal-free metal-organic frameworks (MOFs) with a feature of thermal-differentiated superlattice, in which the localized magnetic heating in periodic spatial distribution makes the spin flip occur at particular active sites, demonstrating a spin-dependent reaction pathway. As a result, the spin-rearranged Co_0.8_Mn_0.2_ MOF displays mass activities of 3514.7 A g_metal_^−1^ with an overpotential of ~0.27 V, which is 21.1 times that of pristine MOF. Our findings provide a new paradigm for designing spin electrocatalysis and steering reaction kinetics.

## Abstract (web summary)

The oxygen evolution reaction in magnetic catalysts is related with their spin configuration. Here, the authors propose a magnetic-stimulation method to rearrange spin electron occupation in thermal-differentiated superlattices.

## Introduction

The massive economic and human development has relied on fossil energies, leading to pronounced degradation of our environment and frequent extreme climate. To address these issues, huge efforts have been directed to water electrolysis to obtain renewable hydrogen, but is facing the dilemma of sluggish reaction kinetics in oxygen evolution reaction (OER)^1,2^. Currently, a grand challenge in industrial application is the lack of inexpensive materials with a low overpotential at high current density and a high durability to replace high-cost iridium- and ruthenium-based electrocatalysts. Therefore, various earth-abundant oxygen catalysts such as transition metal alloys, oxyhydroxides, and oxides have been proposed to enhance OER performance^3–7^. These catalysts mostly toward to the following strategies, namely, constructing various nanostructures, introducing vacancies or defects, and alloying and crystal-phase engineering^8–13^. Generally speaking, current OER mechanisms mainly focus on the thermodynamic process of adsorption and desorption between active sites and intermediates, but spin-related charge transfer and orbital interaction have been unintentionally neglected.

Theoretical investigation discloses that the antiferromagnetic and ferromagnetic are strongly related with the exchange interaction of bonding and antibonding orbitals between magnetic ions and linked organic molecules^14^. Additionally, some reports uncover that the OER performance in magnetic catalysts are intensively interrelated to their spin configuration and exchange interaction^15–17^, because the number of unpaired electrons in four-electron reaction process cannot be conserved, leading to a spin-correlated catalytic activity. These findings inspire us to develop a feasible method to steer catalyst’s spin distribution, which can be used to realize a further breakthrough in OER performance. However, it is generally accepted that the spin state of reactive site has been customized and cannot be easily regulated by traditional synthesis and processing strategies. Therefore, it is difficult to thoroughly understand the correlation between OER mechanism and relative changes in spin configuration. Based on these considerations, we propose a simple magnetic-stimulation approach to realize a spin flip and reconfiguration at reactive site via a coordination mechanism between localized magnetocaloric effect and spin-exchange interaction, finally achieving an unprecedented OER performance.

In order to realize this idea, metal-organic frameworks (MOFs) formed by organic ligands coordinated with magnetic transition metal are designed as periodic thermal conductance units, exhibiting a feature of thermal-differentiated superlattice. Herein, the linked organic molecules with a low thermal conductivity can be used to construct heat-insulating layers, meanwhile coordinated magnetic ions are consciously implanted into interlayers to act as heat conduction regions. When an alternating magnetic field is applied directionally (magnetic stimulation), the magnetic heating is strictly localized around magnetic ions owing to the molecular thermal insulation. As a result, the magnetic exchange interaction can be enforced to drive spin flip and reconfiguration. It is important to note that this design is completely distinguished from conventional catalysts using simple thermal annealing treatment. They are as follows: thermal strain disturbance upon magnetic structure transition can be excluded intentionally; the spin reconstruction triggered by magnetic stimulation can be kept stable for long-term OER application.

In this work, we fabricate bimetallic Co_0.8_Mn_0.2_-MOF materials as thermal-differentiated spintronic catalysts, in which the magnetic heating is periodically distributed to enforce spin-orbital interaction, driving a desirable spin reconfiguration. This spin-reconfigured Co_0.8_Mn_0.2_-MOF demonstrates mass activities of 3514.7 A g_metal_^−1^ at an overpotential of ~0.27 V in OER measurement, which is about two orders of magnitude higher than pristine structure. More interestingly, this spin-reconstructed Co_0.8_Mn_0.2_-MOF can maintain ~95% of its initial activity after 200 h of continuous OER application at a high current density. This is due to the fact that the orbital interaction between this spin-dependent catalyst and the intermediate plays a determining role in the regulation of the rate-limiting step and carrier transfer.

## Results

### Reaction mechanism design and physical characterization

**Fig. 1:**
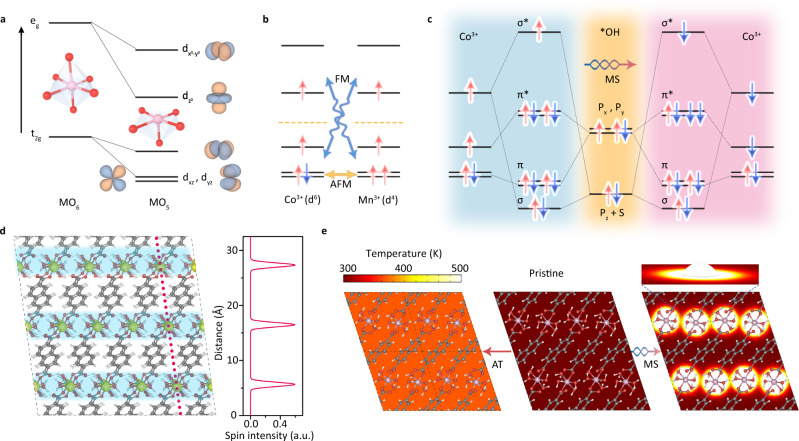
Reaction mechanism design. **a** Electron configuration of 3d orbital. **b** Magnetic exchange sketch. **c** The orbital interaction between Co and *OH under different spin configuration. **d** The spin density distribution of Co_0.8_Mn_0.2_-MOF. **e** The theoretical simulation of heating process between conventional annealing treatment and magnetic stimulation.

Based on the classical catalytic theory, the surface metal site tends to bond with the adsorbed reactants through hybridization between the 2*p* and 3*d* orbitals, which is strongly dependent of e_g_ orbital occupation and spintronic configuration^18^. In the bulk, octahedral ligand field demonstrates that each metal atom is fully coordinated with six oxygen atoms (named as MO_6_) and splits to higher e_g_ and lower t_2g_ orbitals, as shown in Fig. 1a. When these metal atoms are exposed at surface to act as reaction site (named as MO_5_), their catalytic activity will be enhanced because of unpaired 3*d* orbitals to accept the lone-pair electrons of adsorbed intermediates. Take Co and Mn ion for example in Fig. 1b, each t_2g_ and e_g_ orbitals are occupied by several unpaired electrons with single spin, making the unfilled *3d*-shell act as spin-dependent gates to regulate carrier transfer and orbital interaction in catalytic reaction. According to Goodenough-Kanamori rule^19^, the spin coupling interaction between half-filled t_2g_ orbitals usually display an antiferromagnetic (AFM) feature. When empty e_g_ orbitals in higher energy levels are taken into consideration, the electronic hopping between half-filled t_2g_ levels and unfilled e_g_ levels leads to a ferromagnetic (FM) behavior. In this case, one can imagine that if local thermal disturbance is applied to regulate electronic hopping and occupation, this would give rise to a magnetic structure conversion and spin reconfiguration.

Thereby, the orbital interaction between metal active site and intermediates (*OH) is also changed correspondingly, as shown in Fig. 1c. Based on symmetry conservation, the interaction between the Co- *dxz*, *dyz* and *dx*^*2*^*-y*^*2*^ orbitals and the orbitals of the adsorbed *OH are negligible, because these orbitals of metal site cannot mix with the *OH orbitals. According to Hund’s rule, the spin occupation plays a critical role in orbital hybridization, which can be quantitatively reflected by the values of bond order. If spin flip happens at *dz*^*2*^ and *dxy* orbital (marked by different shadows in Fig. 1c) due to a localized thermal contribution, the bond order is increased to 1.5 from 1.0 due to a stronger orbital interaction, benefiting for staring an OER. This is because that the bonding interaction between metal site and reaction intermediate will be enforced as increasing bond order. In this process, unfilled t_2g_ orbitals of catalytic sites can accept valence electrons of the reactant more easily, which is more favorable for the adsorption of *OH to start an OER cycle. In other words, the spin-related OER process can be optimized through a spin reconfiguration. The next consideration is how to realize this proposal.

Subsequently, the organic ligands (2,6-napthalenedicarboxylate acid) with good thermal insulation characteristic (~10^−2^ W m^−1^K^−1^) are proposed to coordinate with magnetic Co and Mn ions (~10^2^ W m^−1^K^−1^) to construct a MOF as shown in Fig. 1d, where the spin charge densities between spin-up and spin-down are symmetrically distributed at interlayer regions (marked by shadows). In this particular case, the magnetic heating generated by alternating magnetic stimulation (MS) can be localized at these magnetic-response regions instead of heating-insulating organic linkers, leading to a thermal-differentiated superlattice. To confirm this feasibility, magnetic heating and heat conduction process in this system is theoretically predicated combining the density function theory with finite element numerical simulation, as shown in Fig. 1e. Different from traditional heating treatment in the whole materials (left panel), the magnetic heating effects are mainly localized at particular magnetic layers (right panel) and the lattice expansion of the whole system is limited effectively. With the increase of MS operation time, the temperature of magnetic layers can reach ~480 K but the remaining regions keep unchanged at room temperature, indicating a typical thermal superlattice feature. Consequently, the spin flip and magnetic structure transition can be successfully achieved at this specific model material.

**Fig. 2:**
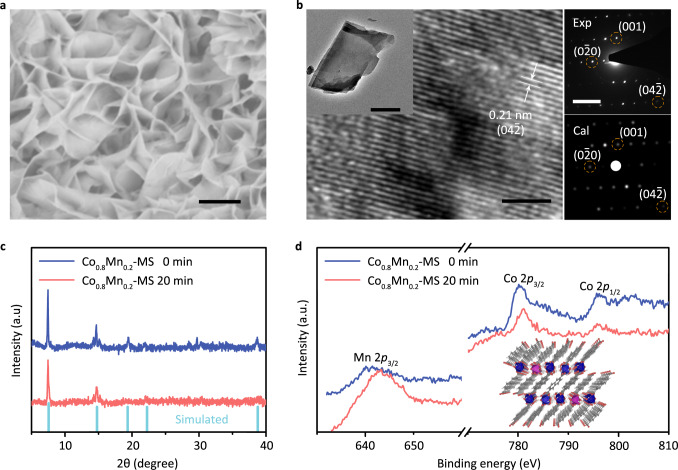
Physical characterization. **a** FE-SEM image of Co_0.8_Mn_0.2_-MOF grown on NF (Scale bar, 5 μm). **b** HR-TEM image of Co_0.8_Mn_0.2_-MOF in the left panel (Scale bar, 2 nm) and the experimental (top) and simulated (bottom) SAED pattern in the right panel (Scale bar, 2/nm). Inset: the TEM image (Scale bar, 1 μm). **c** XRD patterns and **d** XPS high-resolution spectra of Co_0.8_Mn_0.2_-MOF samples with and without MS.

The ultrathin Co_0.8_Mn_0.2_-MOF (taking 2,6-napthalenedicarboxylate acid as the ligand) nanosheet arrays were synthesized onto Ni foam using hydrothermal procedure, which can be confirmed by low magnification EDS image (Supplementary Fig. 1). They were vertically aligned with three dimensional porous texture to serve as a function electrode, which can be clearly observed from scanning electron microscopy (SEM) image in Fig. 2a. The transmission electron microscopy (TEM) (in the inset of Fig. 2b) and the SEM (Supplementary Fig. 2) demonstrate that the average size of each nanosheet is of 130 nm in thickness and 40 μm in width. The selected area electron diffraction (SAED) in the right panel of Fig. 2b displays a pseudotetragonal symmetry (marked by Exp), which is very consistent with the simulated result (marked by Cal). This distinguishable SAED pattern not only confirms the high crystallinity of Co_0.8_Mn_0.2_-MOF but also indicates the structural stability. The high-resolution transmission electron microscopy (HRTEM) in Fig. 2b discloses the resolved lattice fringe is ~2.1 Å, which can be assigned to the ($04\bar{2}$) plane of naphthalene-based MOF with transition metal nodes^20,21^.

Figure 2c shows the X-ray diffraction (XRD) patterns for the Co_0.8_Mn_0.2_-MOFs without and with MS operation (named as Co_0.8_Mn_0.2_, Co_0.8_Mn_0.2_-MS), which are commendably aligned with theoretical simulation. The slight difference between experiment and simulation can be understood by the fact that the theoretical simulation is obtained based on perfect bulk material, nevertheless, the complex nanostructures can lead to a broaden of some XRD peaks owing to the change in geometrical structure factor. Compared to the pristine sample, the main diffraction peaks remain unchanged, indicating no obvious lattice-strain or lattice-expansion can be implanted through MS operation (see detailed analysis about Raman spectra in Supplementary Fig. 3). In addition, some subordination diffractions are depressed because of structural distortion induced by spin reconfiguration, which can be confirmed by the HRTEM image (Supplementary Fig. 4). To reveal the changes in electronic structure, the high-resolution X-ray photoelectron spectroscopy (XPS) results are provided in Fig. 2d. The dominant peaks of Co 2*p*_1/2_, Co 2*p*_3/2_ and Mn 2*p*_3/2_ in pristine Co_0.8_Mn_0.2_-MOF are located at 796.1 eV, 780.6 eV, and 641.3 eV, respectively, implying a strong electronic coupling and the successful insertion of Mn ions. Interestingly, there is only a slight change in the characteristic peak of Co 2p through a MS operation, because of the stable chemical environment of the Co sites. However, the XPS scan in Mn 2*p*_3/2_ core level shifts to a higher energy by 2.1 eV relative to the pristine sample, demonstrating a typical electronic reconstruction in coordination environment. This is because that each Mn site has more unpaired electrons (three) at t_2g_ orbitals than Co site (one), which makes the spin-dependent orbital interaction of Mn-O bonds become more sensitive than that of Co–O bonds. Under MS, the strong d-d Coulomb interaction at Mn atoms can lead to a stronger Jahn-Teller distortion at Mn–O bonds than that of Co–O bonds, thus the corresponding charge transfer between Mn and coordinated O leads to an obvious XPS shift at Mn 2*p*_3/2_ core level. The charge transfer between Mn and coordinated O is triggered by a strong spin–orbital interaction.

**Fig. 3:**
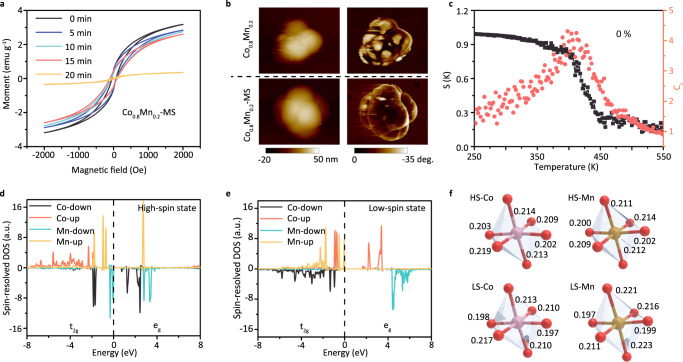
Magnetic structure characterization. **a** Room temperature magnetic hysteresis loops of Co_0.8_Mn_0.2_-MOF with different MS time. **b** The atomic force microscopy topographical (left) and magnetic force microscopy phase (right) image of the Co_0.8_Mn_0.2_-MOF with and without MS. **c** The specific heat *C*_*v*_ and spin structure factor *S*(*k*) as a function of temperature. The spin-resolved PDOS of Co_0.8_Mn_0.2_-MOF with HS **d** and LS **e** configuration. **f** The optimized structure of Co_0.8_Mn_0.2_-MOF with HS and LS configuration.

To obtain insights into the spin-related electronic configuration in Co_0.8_Mn_0.2_-MOF with different MS time, the magnetization as a function of magnetic field (M-H) is investigated in Fig. 3a by a superconducting quantum interference device (SQUID). The pristine sample (0 min) displays a nonlinear hysteresis loop curve, indicating Co_0.8_Mn_0.2_-MOF has a ferromagnetic feature at room temperature owing to nonzero residual magnetization and coercivity. It is important to note that the saturation magnetization intensity and coercivity are decreased simultaneously with the increase of MS time, leading to a partial magnetic structure transition from high-spin (HS) state to low-spin (LS) state. In addition, the magnetic susceptibilities of a function of temperature under field-cooling and zero-field-cooling in Supplementary Fig. 5 disclose that the magnetic ordering temperature dependent of spin-exchange interaction are also changed by MS operation. To corroborate the magnetic structure transition, the magnetic force microscopy (MFM) images of Co_0.8_Mn_0.2_-MOF with and without MS are acquired and compared in Fig. 3b. The topography and MFM images of pristine sample are coincided basically, where the distribution of magnetic domain is one of the most predominant phenomenon in the whole region. After MS operation, the original areas including magnetic domains are contracted correspondingly due to spin reconfiguration, which is in correspondence with measured conclusion of SQUID.

To better understand this physical process, Monte Carlo (MC) simulation using a 13 × 13 × 1 spin matrix is performed to disclose the spin flip and exchange interaction. In this simulation, the spin-related Hamiltonian can be defined as:

$H={J}_{1}\mathop{\sum}\limits_{i,j}{S}_{i}\cdot {S}_{j}+{J}_{2}\mathop{\sum}\limits_{i,j}{S}_{i}\cdot {S}_{j}+{J}_{3}\mathop{\sum}\limits_{i,j}{S}_{i}\cdot {S}_{j}+D\mathop{\sum}\limits_{i}{S}_{i,x0}^{2}$, where *J*_*1*_, *J*_*2*_, and *J*_*3*_ corresponds to the first-, second-, and third-nearest magnetic coupling strength, respectively. *S*_*i*_ and *S*_*j*_ are the net spin, *S*_*i,x*0_ and *D* is the component of *S*_*i*_ along the easy-axis direction and single-ion anisotropy parameter. When a thermal equilibrium is reached at a given temperature, the specific heat can be considered by ${C}_{{{{{{\mathrm{v}}}}}}}=({E}^{2}-{E}^{2})/{K}_{B}{T}^{2}$, here *E* is the total energy of the system. As a result, the specific heat *C*_v_ and spin structure factor *S*(*k*) as a function of temperature is acquired in Fig. 3c. It can be observed that the systematic temperature plays a determining role in driving spin flip and reconfiguration, especially in the range of Curie temperature. According to Heisenberg model^22^, the stability conversion between HS and LS configuration can be realized under an appropriate temperature condition, which gives rise to a spin reconstruction and reaches a new equilibrium. Interestingly, when the thermal strain is considered into the whole region, the temperature of generating spin reconfiguration will be enhanced (Supplementary Fig. 6).

To better understand this spin reconfiguration process, the spin-resolved projected density of states (PDOS) of Co_0.8_Mn_0.2_-MOF with HS and LS configuration are shown in Fig. 3d, e, whose bandgap is ~0.33 eV (HS state) and 0.55 eV (LS state), respectively. The electronic hoping between half-filled t_2g_ orbitals of Co and unfilled e_g_ orbitals of Mn is responsible for magnetic enhancement, which is enhanced as reducing bandgap. After considering magnetic heating effect, the conduction region (unoccupied Co-e_g_ orbital) begin to far away from valence band (half-filled Mn-t_2g_ orbital), which makes it transform into LS state because of restraining electronic hopping. In this case, the exchange interaction between Co-t_2g_ and Mn-t_2g_ orbital becomes one of the most predominant factors, resulting in a LS state. The spin reconfiguration can lead to a structural distortion and charge transfer through a spin-orbital interaction, as shown in Fig. 3f. The Bader charge of Co and Mn is changed to 7.66, 11.48 (HS state) from 7.76, 11.58 (LS state) and the bond lengths of Co-O and Mn-O are also stretched asymmetrically. From here we can see that the spin-related electronic structure transition may provide a new feasibility into regulating OER mechanism.

### Practical application and catalytic mechanism of OER

**Fig. 4:**
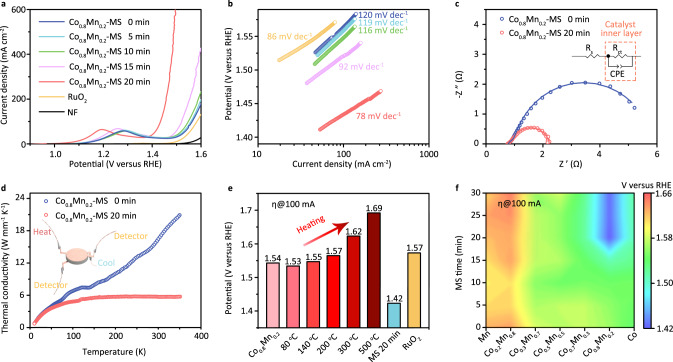
Practical application of OER. **a** Polarization curves and **b** Tafel plots of the Co_0.8_Mn_0.2_-MOF with different times of MS. **c** EIS curves and **d** lattice thermal conductivity of the Co_0.8_Mn_0.2_-MOF with and without MS. Inset: the equivalent circuit. **e** The potential of OER for Co_0.8_Mn_0.2_-MOF at 100 mA cm^−2^ with different annealing treatment temperature and 20 min MS as reference. **f** The mapping results of the potential at 100 mA cm^−2^ for Co_x_Mn_1-x_-MOF with different ratios of Co/Mn and MS time.

To disclose the contribution of spin reconfiguration, the linear sweep voltammetry curves of Co_0.8_Mn_0.2_-MOF electrodes with various MS time are obtained in Fig. 4a. It is interesting to note that the anodic peaks in the OER polarization curves at 1.2–1.3V versus RHE can be attributed into the oxidation of Co ions^23,24^. As a general feature, the OER performance of our prepared Co_0.8_Mn_0.2_-MOF is completely superior to the commercial RuO_2_ electrode especially at high current density. Because, the octahedral crystal field theory discloses that the electronic configuration for Mn ion and Co ion are t_2g_^3^e_g_^1^ and t_2g_^5^e_g_^1^, in which the filling t_2g_ and e_g_ orbitals play a critical role in determining their exchange interaction as shown in Fig. 1b. When the magnetic ions bond to coordinated anions, the metal sites with empty t_2g_ orbital can be used to accept the valence electrons of conjugated O atoms that can donate into the bonding orbital (σ and π) to construct the Mn–O and Co–O bonds. Therefore, the magnetic response of Mn ion with more unpaired t_2g_ electrons is better than Co ion. In order to obtain better exchange interaction, the concentration of Mn ion should be lower than that of Co ion. Compared to pristine MOF sample, the anodic peaks of the Co_0.8_Mn_0.2_-MOF with 20 min MS are obviously shifted to a lower potential by 88 mV, which indicates a lower rate-limiting potential barrier and more active sites due to spin reconfiguration (Supplementary Fig. 7)^25^. The detailed correlation between potential shift and MS time in Supplementary Fig. 8 demonstrates that the potential at 200 mA cm^−2^ sharply decrease along with spin reconfiguration at initial 15 min and then slowly tend to an equilibrium state with 1.46 V potential. The optimal MS time is about 20 min, because spin flip prefers to occur at this operating time window. After finishing spin reconfiguration, the OER performance slowly tends to an unchanged behavior. It is important to note that the electrochemical (EC) behaviors of pure nickel foam (NF) and the traditional CoMn oxyhydroxide sample cannot be affected by MS, as shown in Supplementary Figs. 9 and 10. In addition, Tafel plots (Fig. 4b) are provided to furthermore disclose the spin-related catalytic process. Relative to pristine sample (120 mV dec^−1^), the Tafel slopes are decreased to 119, 116, 92, and 78 mV dec^−1^ with increasing MS time, implying a possible change in the activation barrier because of spin-dependent orbital interaction between the active sites and intermediates.

The corresponding electrochemical impedance spectra (EIS) in Fig. 4c demonstrate that the conductivities are also strongly related with spin flip and reconstruction. The remarkable reduction in charge transfer resistance (Rct) further demonstrates that spin reconfiguration not only provides a higher catalytic activity but also benefits for charge transfer^26,27^. Notably, the Co_0.8_Mn_0.2_-MOF with 20 min MS displays an excellent structural stability with ~5% decay of the initial current density after 200 h OER test, as shown in Supplementary Fig. 11, and the Faradaic efficiency can maintain at 98.2 ± 1.3%^28^. The original morphology, chemical composition, and the organic linker remain unaffected after the long-time stability testing (Supplementary Figs. 12–15)^29^. In addition, the spin configuration induced by MS can remain stable, which can be confirmed by the unchanged SQUID measurement before and after OER test in Supplementary Fig. 16. To understand the relationship between thermal disturbance and spin reconfiguration, the temperature dependence of thermal conductivity is shown in Fig. 4d and Supplementary Fig. 17. We can find that the value of pristine sample is obviously higher than that of sample with MS operation, because electron–phonon interactions can be reflected by spin configuration. In the HS state, the magnetic behavior of pristine sample is rigid and cannot be affected by the spin state of the high-energy carrier, which leads to a single scattering event (Supplementary Fig. 18). When it transforms into LS state, the magnetic ordering becomes no longer rigid that can be regulated randomly. Similar to the Kondo effect^30,31^, the multiple electron-phonon scattering is responsible for a lower thermal conductivity.

To reveal the difference between our strategy and traditional thermal treatment, the potentials at 100 mA cm^−2^ as a function of Co_0.8_Mn_0.2_-MOF with various annealing temperatures are compared in Fig. 4e. It can be found that the potential is slightly decreased from 1.54 V (pristine) to 1.53 V (80 °C annealing treatment) and then increase sharply with annealing temperature, which are all larger than the sample with MS. In traditional annealing treatment, the lattice expansion makes the temperature of magnetic phase transition is enhanced due to the overall heating method (as discussion in Fig. 3). Note that they can easily relax to initial states after removing heating source, which can be confirmed by the temperature-resolved Raman spectra in (Supplementary Fig. 19). When the annealing temperature is further enhanced, the crystalline structure will be destroyed, leading to a negative OER performance (Supplementary Fig. 20). Finally, the catalytic activity of various Co_x_Mn_1-x_-MOFs under different MS time are compared in Fig. 4f (the corresponding experimental details are displayed in Supplementary Figs. 21 and 22). The high MS-response occurs at Co_0.8_Mn_0.2_-MOF and these changes cannot be observed in pure Co-MOF and Mn-MOF sample. This is because that the energy difference between HS and LS configuration closes to zero, thus leads to a paramagnetic behavior (Supplementary Fig. 23). Non-equivalent magnetic moment between Co (1μ_B_) and Mn ions (3μ_B_) in CoMn-MOF system can lead to a stronger exchange interaction to trigger spin flip and reconfiguration. That is why we choose this system in this work.

**Fig. 5:**
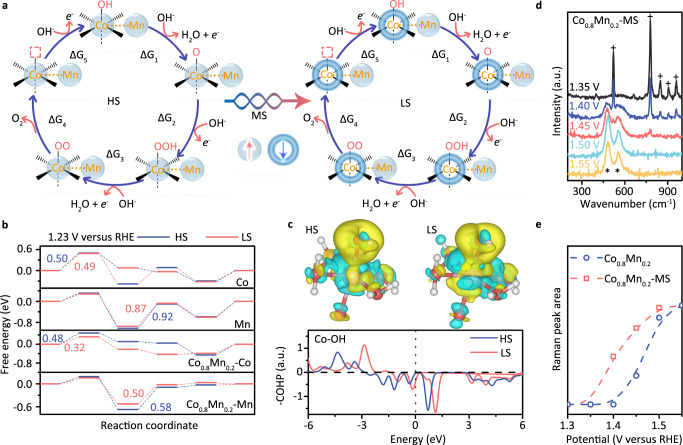
Correlation of OER mechanism with different spin configuration. **a** The OER process of Co_0.8_Mn_0.2_-MOF with HS and LS configuration. **b** Free energies of OER steps at different reactive sites with HS and LS configuration. **c** The top panel: the charge density difference of adsorbed *OH onto Co site. The bottom panel: the crystal orbital Hamilton population (COHP) of adsorbed *OH onto Co site. **d** Raman spectra of the Co_0.8_Mn_0.2_-MS MOF sample with applying different potentials. **e** The relative Raman intensity as a function of applied potentials.

To elucidate the spin-related catalytic mechanism, the reaction pathway including one thermodynamic step (ΔG5) and four EC steps are displayed in Fig. 5a, with Mn-Co(OH) as a unified starting point (ΔG1). To simulate the magnetic heating contribution onto catalytic activity via Co-Mn exchange interaction, the key difference between HS and LS state can be considered as that the spin configuration of Co site are flipped and then to react with intermediates. Therefore, the free energy differences (ΔG) between the two isomeric reactants and rate-limiting barrier are regulated owing to the spin-dependent orbital hybridization^1^. For the Co-MOF, the OER follows the HS pathway (the first panel in Fig. 5b), because adsorption of *OH with HS interaction in step 1 acts as the potential-determining step (0.50 eV), which is slightly higher in energy than that of LS intermediates (0.49 eV). For the Mn-MOF (the second panel in Fig. 5b), the OER still prefers to choose LS pathway but the rate-limiting reactant occurs at step 3. The formation of *OOH at Mn site requires more energy to be realized (0.87 eV in LS pathway and 0.92 eV in HS pathway), which are obviously higher than the other reactants. We can conclude that the catalytic activity of OER is authentically related with spin configuration of reactive site.

When magnetic exchange interaction is considered into Co_0.8_Mn_0.2_-MOF, the determining activation energies at Co site (the third panel in Fig. 5b) and Mn site (the fourth panel in Fig. 5b) are obviously lower than that of corresponding reactive site in pure Co-MOF and Mn-MOF. More importantly, in the Co_0.8_Mn_0.2_-MOF, the differences of relative stability between HS and LS configuration at rate-limiting step are enlarged due to a stronger spin-orbital interaction^14^. For example, the potential-determining step is identified to the adsorption of *OH onto Co site and the corresponding OER overpotential is decreased to 0.32 eV (LS) from 0.48 eV (HS). This selectivity of the reaction pathway in LS configuration also occurs at Mn site, as shown in the fourth panel in Fig. 5b. According to the lowest principle of energy, in the Co_0.8_Mn_0.2_-MOF, the Co site with LS configuration plays a critical role in determining OER performance.

To disclose the correlation between spin configuration and free energy, the charge density difference of adsorbed *OH onto Co site are calculated in the top panel of Fig. 5c. The yellow isosurface corresponds to an electron increase zone, and the blue one is an electron depletion zone. When *OH is attracted onto catalytic surface, the unpaired O-*2p* orbitals are more likely to hybridize with LS Co -*3d* orbital. Compared to HS state, the electron increase zone (yellow) in LS state is extended to the coordinated Co–O bonds, which makes the e_g_ electron occupancy of Co ion reduce to 2.97 (LS) from 2.99 (HS). The metal-oxygen bonds exhibit a mixed ionic-covalent characteristic owing to the energetic similarity (covalency) and spatial overlap between Co-*3d* orbital and O-*2p* orbital, which plays a critical role in determining catalytic activity. To confirm this point, the crystal orbital Hamilton population (COHP) is calculated to compare the bonding character between LS and HS state^1,15^. Different from the Co–OH bonding contribution in HS state, a stronger antibonding state appear at Fermi level, confirming that the electrons from Co-*3d* orbital partially transfer to the unfilled O-*2p* orbital. The stability of Co–OH bond in LS state is lower than that of HS state, thus leading to a lower reaction activation energy.

To further uncover the spin-dependent catalytic activity, a series of in situ Raman spectra as a function of the applied potential during an oxidation sweep are acquired in Fig. 5d. The spectral feature with applied potential discloses that two additional broad Raman peaks (marked by *) at 477 and 623 cm^−2^ begin to appear at 1.4 V (Supplementary Fig. 24), which can be attributed to the formation of some intermediates (*O) at Co_0.8_Mn_0.2_-MOF sample surface. This Raman response is similar with previous reports about the vibrational behavior of Co–O bonds after the electrodes were pretreated at the various voltage states^32–34^. With increasing potential, fingerprint peaks of Co_0.8_Mn_0.2_-MOF sample with 20 min MS (marked by +) are submerged by intermediate signals and meanwhile some oxygen bubbles can be observed on the electrodes. These spectral changes inspire us to evaluate the OER activity through the ratio of the Raman intensity of intermediates to the relative Raman mode of catalyst^28^: *R* = $\int (S_{1}+S_{2})/\int S_{{{{{\rm{max }}}}}},$ where the *S*_1_ and *S*_2_ are the two additional broad Raman peaks (marked by *), *S*_max_ is the maximum peak areas of *S*_1_ and *S*_2_. To acquire the detailed Raman behavior of intermediates and catalysts, the relative intensity as a function of applied potential is assessed in Fig. 5e. For Co_0.8_Mn_0.2_-MOF with MS, the *R* remains value of zero for the forward sweep below 1.4 V, which indicates that the intermediates play a negligible factor and the dominate signals are originated from catalysts. With the increase of applied potential, the *R* values begin to increase sharply at ~1.45 V and finally approach a maximum value at ~1.65 V, disclosing a typical OER behavior. It is interesting to note that this opening behavior is blue-shifted to 1.5 V in pristine sample (blue curves, see detailed Raman spectra in Supplementary Fig. 25) and relative intensity (R) is also smaller than that of Co_0.8_Mn_0.2_-MOF with MS. These in situ Raman spectra can be used to explain why the sample with MS can display an outstanding OER performance^35,36^.

## Discussion

In this work, we have demonstrated that the localized magnetic heating induced by MS can lead to a spin flip and reconfiguration, through designing thermal-differentiated superlattices. More interestingly, charge transfer and orbital interaction between the reactive sites and the reactants display a spin-dependent reaction kinetics, making the catalyst demonstrate mass activities of 3514.7 A g_metal_^−1^ at an overpotential of ~0.27 V. Our findings provide a new insight for boosting OER performance in the fields of spin-related electrocatalysis.

## Methods

### Preparation of ultrathin CoMn-MOF electrode

All chemicals were of analytical grade and used as received without further purification. In a typical process, 8 mg cobaltous nitrate and 2 mg manganous nitrate (50 wt.% in H_2_O) were dissolved into 10 mL of deionized water and the solution was vigorously stirred about 1 h to ensure adequate dispersion, followed by further sonication for 45 min under the same conditions. Meanwhile, 10 mg of organic ligand 2,6-naphthalenedicarboxylate acid was dispersed in the 2 mL of ethanol solvent and then was injected into the above solution using a pipette at a rate of 0.2 mL min^−1^ and the mixture was stirred under the high-purity argon (Ar 99.999%) current protection for 30 min to form a homogeneous solution at room temperature. Thereafter, the pre-cleaned nickel foam (NF) substrate was vertically aligned in the above precursor solution. Finally, the obtained suspension within NF was transferred to 15 mL Pyrex tube and then heated in an oven at 85 °C for 20 h. After naturally cooling down to room temperature, the NF was sonicated for 1 min in an ice bath by a sonicator and washed several times with deionized water, and dried under an atmosphere of Ar at 60 °C overnight. The mass of cobaltous nitrate varied from 2, 3, 5, 7 to 8 mg and the manganous nitrate varied from 8, 7, 5, 3 to 2 mg at the same time. The resultant products were named as Co_*x*_Mn_1-*x*_-MOF (*x* = 0.2–0.8), where *x* referred to the percent of Co and Mn source mass. Pure Co-MOF or Mn-MOF sample was also prepared via the same route only without the involvement of each other. The as-prepared MOFs were further placed into an external high-frequency alternating magnetic field using commercial induction set-up (Honghu, HD-20) operated at 150 kHz as the source of magnetic stimulation with a root mean square amplitude in the range 0.1T for different periods of 5, 10, 15, and 20 min to obtain various Co_*x*_Mn_1-*x*_-MOF-MS samples. The amount of MOF deposited on NF can be obtained by calculating the quantity difference between NF and MOF/NF (Supplementary Fig. 26).

### Electrochemical measurements of pristine and magnetization-stimulated MOFs

The EC measurements were performed using an EC workstation (CHI 660D) at room temperature. A standard three-electrode electrolyte configuration with 1 M KOH solution was used in all tests, with a graphite rod (Alfa Aesar, 99.9995%) as the counter electrode, commercial Ag/AgCl (saturated KCl) as the reference electrode, and NF with the geometric area of 1 cm^2^ loaded with different samples as the working electrodes. For a comparison, commercial RuO_2_ powders were loaded on pretreated NF with a loading mass of 2.0 mg cm^−2^ via drop-casting of a catalyst ink. The homogeneous catalyst ink was prepared by mixture of 1 mL ethanol/water (5:1 v/v) solution, 10 mg of catalyst powder and 100 μL Nafion solution (5 wt.%, Du Pont). Before the OER kinetics measurements, the electrolyte was bubbled with high-purity oxygen for at least 20 min to ensure the H_2_O/O_2_ equilibrium. All the data were presented with iR correction through reversible hydrogen electrode (RHE) calibration. The linear sweep voltammetry (LSV) was recorded with the scan rates of 5 mV s^−1^. The external potentials [E(Ag/AgCl)] were measured against the Ag/AgCl electrode as reference which could convert to the potential versus RHE by using the Nernst function: ${{{{{\rm{E}}}}}}({{{{{\rm{RHE}}}}}})={{{{{\rm{E}}}}}}({{{{{\rm{Ag}}}}}}/{{{{{\rm{AgCl}}}}}})+{{{{{{\rm{E}}}}}}}^{0}({{{{{\rm{Ag}}}}}}/{{{{{\rm{AgCl}}}}}})+0.059{{{{{\rm{PH}}}}}}$, where ${{{{{{\rm{E}}}}}}}^{0}({{{{{\rm{Ag}}}}}}/{{{{{\rm{AgCl}}}}}})$ is the standard electrode potential of Ag/AgCl reference electrode. The Tafel plots were acquired by re-plotting the polarization curves as overpotential (*η*) vs. log current (log *j*) and were estimated by fitting the linear portion of the Tafel plots to the Tafel equation (*η* = *b* log(*j*)  + a), where *η* is overpotential, *j* is the current density, and *b* is the Tafel slope. To evaluate the EC stability, the catalyst was tested by the potential retention performance for 200 h at a current of 100 mA cm^−2^. The EIS were recorded under the following conditions: AC potential perturbation 5 mV, frequency ranges 100 kHZ to 0.01 Hz, and open circuit. To determine the double-layer capacitance (*C*_dl_), the cyclic voltammograms were tested at various scan rates (10, 20, 40, 60, 80, 100 mV s^−1^) between 1.06 V and 1.10 V (vs. RHE)^25,37^.

### Density functional theory calculation

The theoretical simulation was conducted based on Vienna ab initio simulation package (VASP), with Perdew-Burke-Ernzerhof (PBE) and generalized gradient approximation (GGA)^38,39^. The plane-wave cutoff energy was 460 eV and the Monkhorst-Pack k-points grid was set to be 7 × 7 × 1. The vacuum space thickness of surface slab was 20 Å and the atomic forces were converged to be 0.01 eV/Å, which were tested to be well converged.

## Supplementary information

Supplementary Information

## Data Availability

The data described in this paper are available from the authors upon reasonable request.
