# Supplementary material for: Spin-state reconfiguration induced by alternating magnetic field for efficient oxygen evolution reaction
Source: Nat Commun. 2021 Aug 10;12:4827. doi: 10.1038/s41467-021-25095-4 (PMC8355122; doi:10.1038/s41467-021-25095-4)
Supplement: Supplementary file 1 — Supplementary Information [file 41467_2021_25095_MOESM1_ESM.pdf]

Supplementary Information for Manuscript

Spin-sate reconfiguration induced by alternating magnetic field for efficient oxygen evolution reaction

Zhou et al.

Supplementary Figures

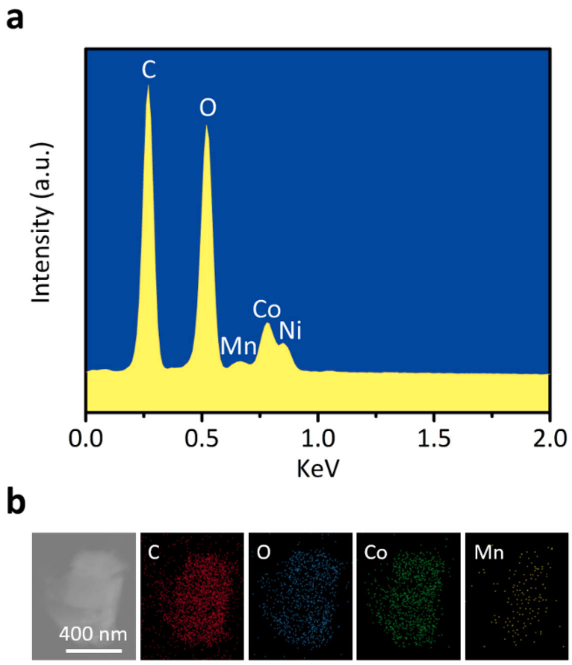

Supplementary Figure 1. (a) EDS spectrum and (b) EDS mapping of the $Co_{0.8}Mn_{0.2}$-MOF. No noticeable impurities are introduced unintentionally in the preparation process. The detected Ni element is derived from the NF substrate. The as-prepared nanostructures are mainly composed of Co and Mn elements at approximate ratio of 4:1.

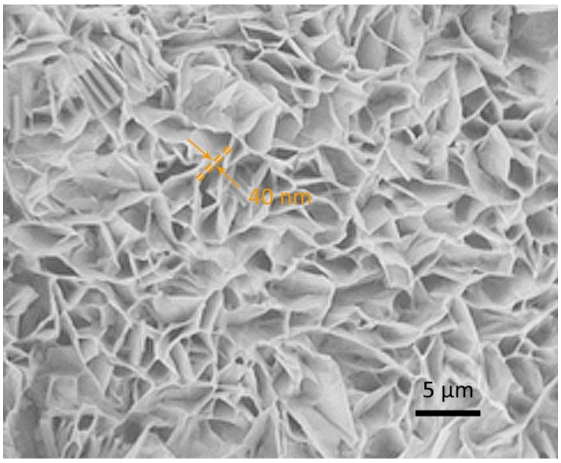

Supplementary Figure 2. The SEM images of the $Co_{0.8}Mn_{0.2}$-MOF. The average thickness of $Co_{0.8}Mn_{0.2}$-MOF nanosheet is of 130 nm in thickness.

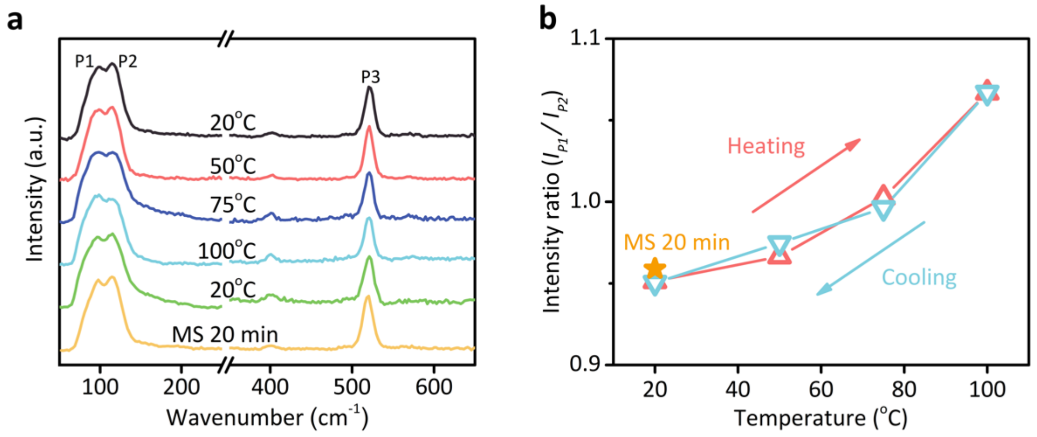

Supplementary Figure 3. (a) Raman spectra of the $Co_{0.8}Mn_{0.2}$-MOF sample with different annealing treatment. (b) The P1 and P2 mode ratio of the $Co_{0.8}Mn_{0.2}$-MOF sample. As additional evidences in Supplementary Figure 3, the detailed Raman spectra are provided to exclude thermal expansion contribution in magnetic stimulation. It can be found in Supplementary Figure 3(a) that relative intensities of P1 and P2 mode are changed as lattice-expansion from 20 °C to 100 °C, and which can recover to initial state (green lines) after removing heat source. It is interestingly to note that the Raman fingerprint of $Co_{0.8}Mn_{0.2}$-MOF with MS is obviously different from that of lattice-expansion. In addition, the slight spectral differences of $Co_{0.8}Mn_{0.2}$-MOF with and without MS can be attributed to the structural distortion induced by spin reconfiguration. In order to distinguish this factor from thermal expansion, the relative intensity of P1 and P2 mode as a function of annealing treatment is compared in Supplementary Figure 3(b). The signal of $Co_{0.8}Mn_{0.2}$-MOF with MS is obviously different from pristine sample with lattice-expansion, which can be directly used to distinguish MS

contribution from simple thermal expansion. This detailed analysis about Raman spectra is consistent with the XRD pattern in Fig. 2c.

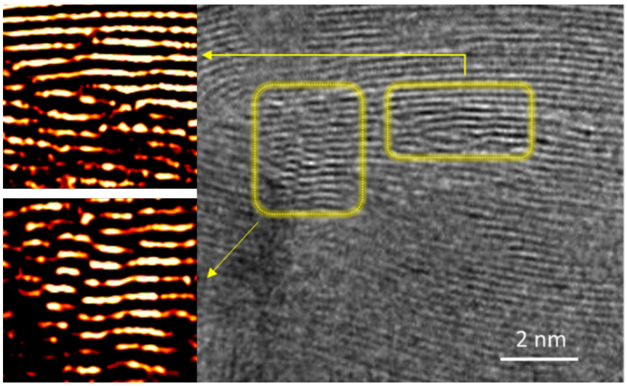

Supplementary Figure 4. HR-TEM image of $Co_{0.8}Mn_{0.2}$-MOF after MS 20 min. In order to disclose the structural distortion induced by magnetic stimulation, the HRTEM images of $Co_{0.8}Mn_{0.2}$-MOF with 20 min MS are provided. Compared to the pristine sample in Fig. 2b, the slight distortion can be observed in some parts of the HRTEM image after magnetic stimulation, but they still maintain the original arrangement and electron conjugated system. This slight atomic distortion can be attributed to the spin electronic difference between Co ($t_{2g}^5e_g^1$) and Mn ($t_{2g}^3e_g^1$). It is generally accepted that difference in t_{2g} orbital occupation can lead to a different Jahn-Teller distortion between the Mn-O and Co-O coordination octahedral. When spin reconfiguration is realized by magnetic stimulation, an obvious disordered atomic arrangement on the individual surface can be obtained, which is responsible for the slight changes in XRD patterns.

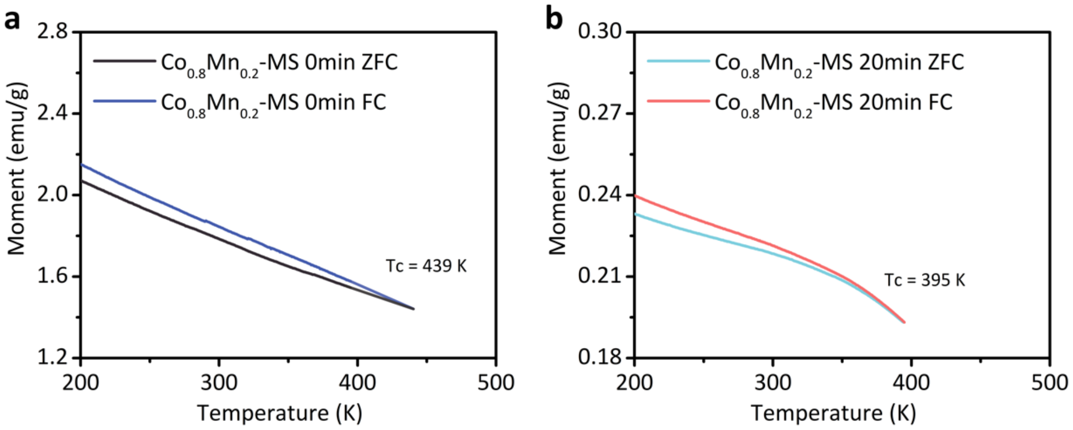

Supplementary Figure 5. Temperature dependence of the magnetic susceptibility measured at 500 Oe external magnetic-field cooling (FC) and zero magnetic-field cooling (ZFC) without (a) and with (b) MS.

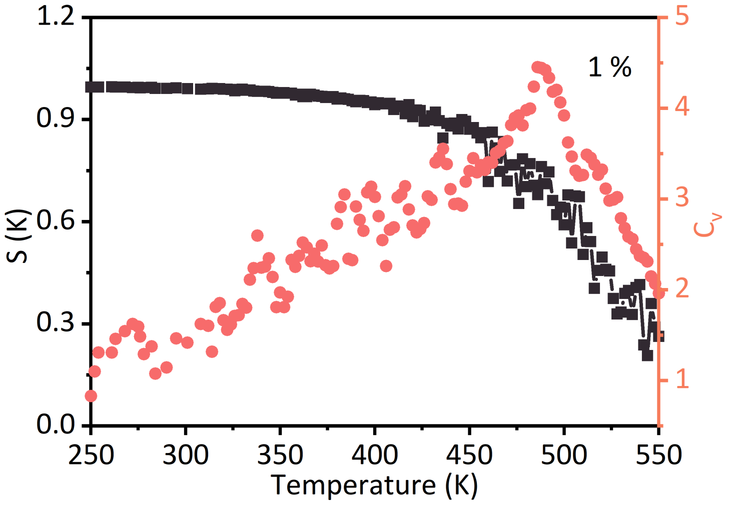

Supplementary Figure 6. The specific heat C_v and spin structure factor $S(k)$ as a function of temperature for $Co_{0.8}Mn_{0.2}$-MOF sample with 1% thermal strain.

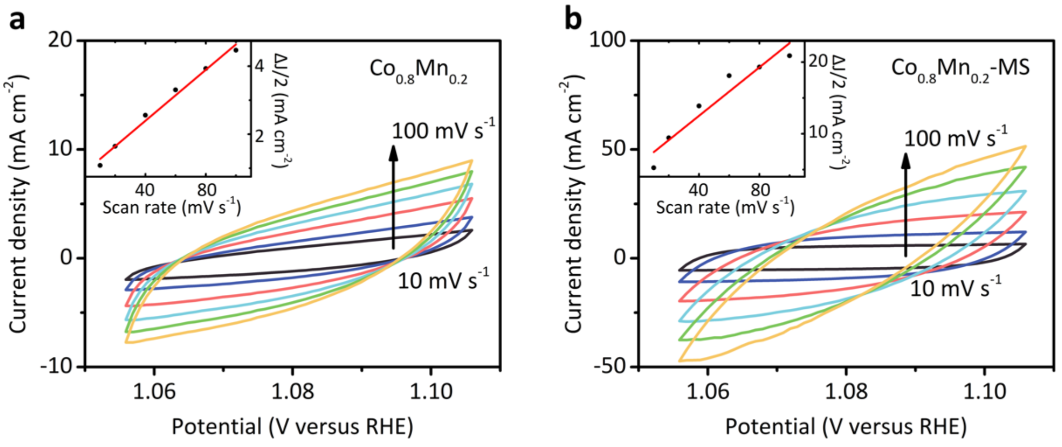

Supplementary Figure 7. Cyclic voltammetry curves of $Co_{0.8}Mn_{0.2}$-MOF (a) without and (b) with MS at various scan rates. Inset： the corresponding electrochemical double layer capacitance plots. To assess the electrochemical active surface area (ECSA), double layer capacitance (C_{dl}) of the catalyst was measured by a simple cyclic voltammetry method to calculate roughly the value of ECSA. The current densities at the selected potentials from the regions of no Faradaic processes were used to get the fitting curve, the slope of which is considered as the C_{dl}. To measure electrochemical double-layer capacitance (C_{dl}), the potential was swept six times at each scan rate (10-100 mV/s) in the scan range from 1.06 to 1.10 V vs. RHE. Capacitive currents were measured in a potential range where no faradic processes happen. The measured capacitive current difference (ΔJ/2) at 1.08 V vs. RHE was plotted against scan rate and specific capacitance was determined from the slope of the linear fitting. The C_{dl} values for $Co_{0.8}Mn_{0.2}$-MOF with and without MS are calculated to be 38 and 169 mF·cm^{-2}, respectively. The specific capacitance is converted into an electrochemical surface area (ECSA) using the specific capacitance value for a flat standard with 1 cm^2 of real surface area. We used the specific capacitance (20–60 μF

cm^{-2}) of 40 μF cm^{-2} here to calculate the ECSA according to the Eq:

$$\mathrm{ECSA} = \frac{C_{\mathrm{dl}}}{40\,uF/cm^2}\,cm^2_{\mathrm{ECSA}}.$$

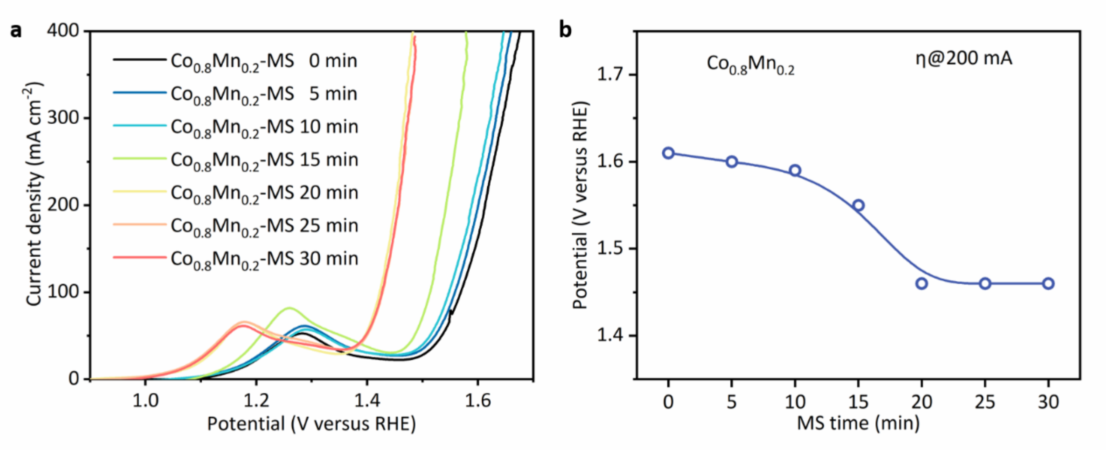

Supplementary Figure 8. (a) Polarization curves of the $Co_{0.8}Mn_{0.2}$-MOF with different times of MS. (b) The potential of OER for $Co_{0.8}Mn_{0.2}$-MOF at 200 mA cm^{-2} with different MS time. It is interesting to note that the anodic peaks in the OER polarization curves at 1.2-1.3 V versus RHE can be attributed into the oxidation of Co ions. Compared to pristine MOF sample, the anodic peaks of the $Co_{0.8}Mn_{0.2}$-MOF with 20 min MS are obviously shifted to a lower potential by 88 mV, which indicates a lower rate-limiting potential barrier and more active sites due to spin-reconfiguration. The detailed correlation between potential shift and MS time in Supplementary Figure 8(b) demonstrates that the potential at 200 mA cm^{-2} sharply decrease along with spin reconfiguration at initial 15 min and then slowly tend to an equilibrium state with 1.46 V potential. When the magnetic stimulation is applied, the spin reconfiguration happens consequently and the reaction rate-limiting potential barrier is reduced correspondingly, leading to a higher catalytic activity. The optimal MS time is about 20 min, because spin flip prefers to occur at this operating time window. After finishing spin reconfiguration, the OER performance slowly tends to an unchanged behavior.

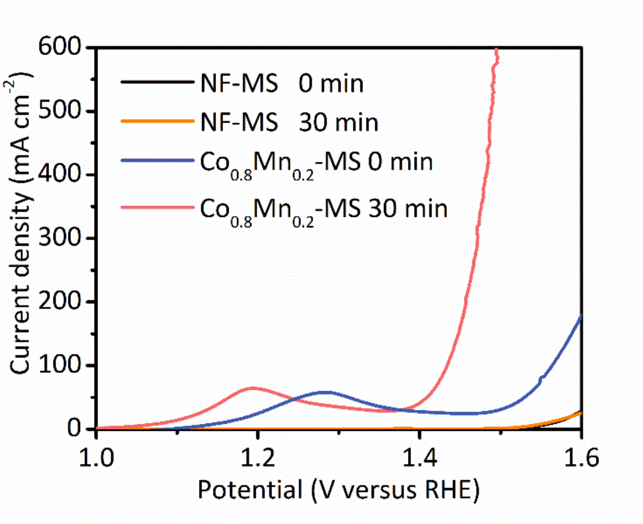

Supplementary Figure 9. Polarization curves of the nickel foam (NF) and $Co_{0.8}Mn_{0.2}$-MOF with/without MS. The electrochemical behaviors of NF with/without magnetic stimulation were conducted and compared in Supplementary Figure 9. Different from substantial enhancement in OER performance for $Co_{0.8}Mn_{0.2}$-MOF, there is no observable change in pure NF with and without magnetic stimulation.

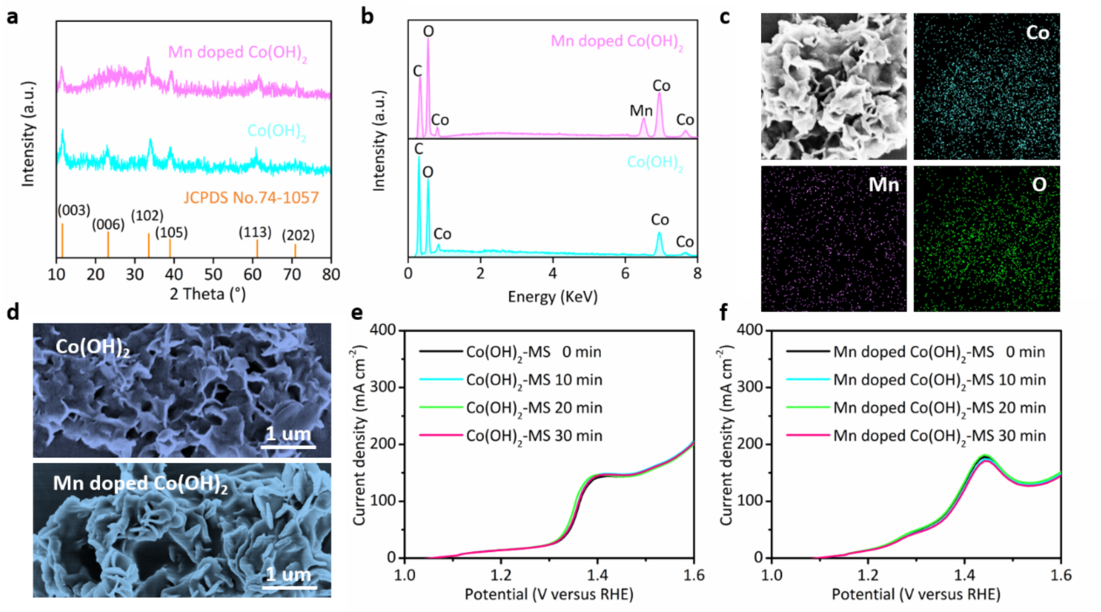

Supplementary Figure 10. The XRD patterns (a) and EDS spectra (b) of $Co(OH)_2$ and Mn doped $Co(OH)_2$. (c) EDS mapping of Mn doped $Co(OH)_2$ sample, indicating the successful preparation of Mn doped $Co(OH)_2$ and homogeneous distribution of Co, Mn and O elements. (d) SEM images of the $Co(OH)_2$ and Mn doped $Co(OH)_2$. (e) Polarization curves of pristine $Co(OH)_2$ and (f) Mn doped $Co(OH)_2$ with different MS times, respectively.

The tested results disclose that the OER polarization curves of $Co(OH)_2$ sample with and without doping Mn cannot be changed as MS time, which is completely different from $Co_{0.8}Mn_{0.2}$-MOF sample. Therefore, we can conclude that the spin reconfiguration cannot occur at the $Co(OH)_2$ sample.

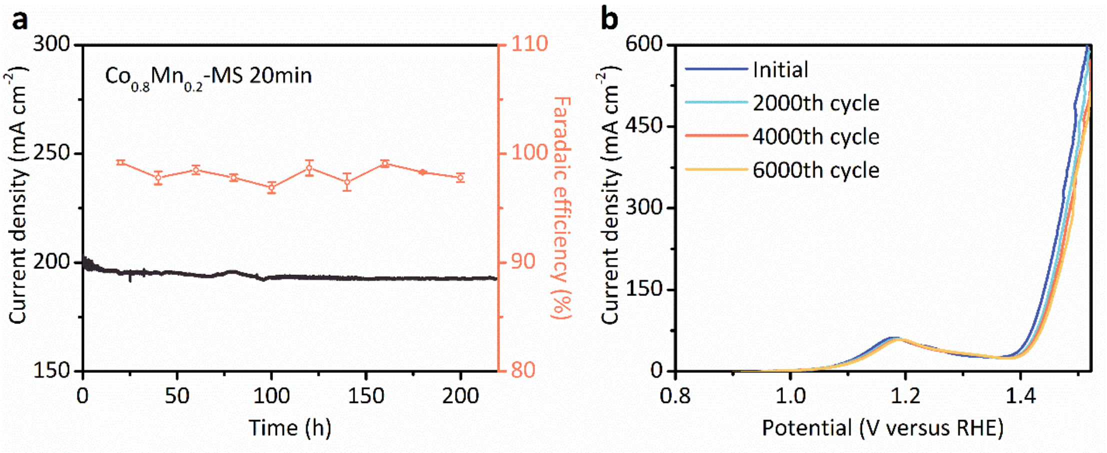

Supplementary Figure 11. (a) The I–t curve displays no significant change even after 200 h cycling, and the Faradaic efficiency can maintain at 98.2±1.3%, indicating the constant OER. (b) Polarization curves of the $Co_{0.8}Mn_{0.2}$-MOF with 20 min MS before and after 6,000 cycles.

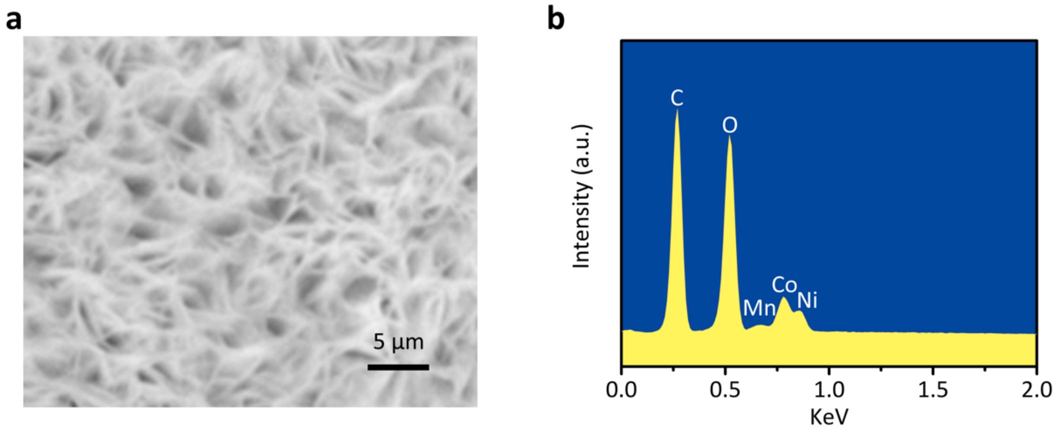

Supplementary Figure 12. (a) FE-SEM image and (b) EDX spectrum of the $Co_{0.8}Mn_{0.2}$-MS under long-term stability testing. There are no discernible changes in the local morphology, crystal structure and chemical composition of $Co_{0.8}Mn_{0.2}$-MS.

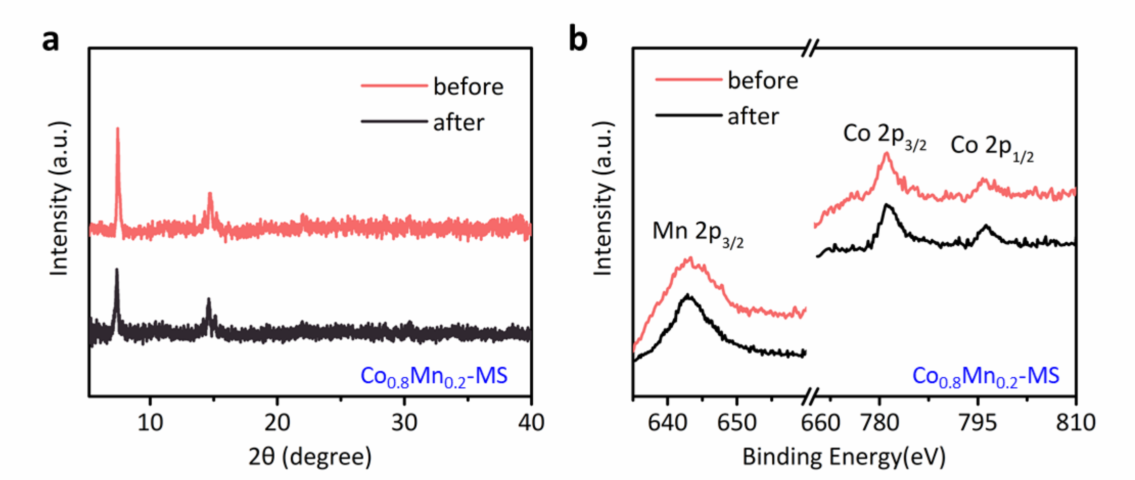

Supplementary Figure 13. The (a) XRD and (b) XPS spectra of $Co_{0.8}Mn_{0.2}$-MS nanosheets before and after OER test. In order to better confirm the stability, additional XRD and XPS before and after OER test are also provided in Supplementary Figure 13. We can see that the XRD and XPS spectra of $Co_{0.8}Mn_{0.2}$-MS display a coincident behavior before and after OER test, indicating no crystal structure destruction occurs.

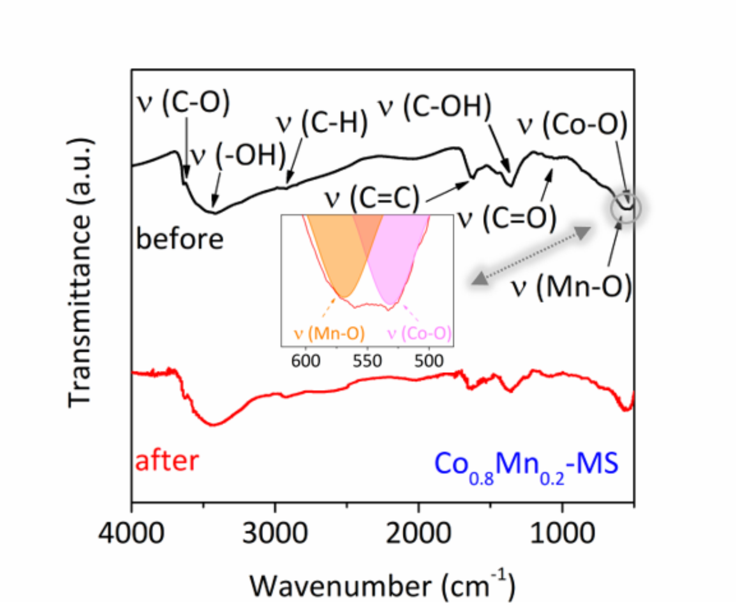

Supplementary Figure 14. FT-IR spectra of the $Co_{0.8}Mn_{0.2}$-MS nanosheets before and after OER test. In order to prove the stability of the organic linker of $Co_{0.8}Mn_{0.2}$-MS, the Fourier transform infrared spectroscopy (FT-IR) before and after OER test are conducted and shown in Supplementary Figure 14. Apart from the aromatic C=C skeletal vibration of the sp^2 domains (1632.4 cm^{-1}), the FT-IR spectra of $Co_{0.8}Mn_{0.2}$-MS with and without stability test both display the presence of oxygenated functional groups near 1067.1 cm^{-1} (carbonyl C=O in carbonyl, and carboxyl moieties), 1366.3 cm^{-1} (hydroxyl C−OH), 2915.8 cm^{-1} (C−H), 3429.6 cm^{-1} (structural O−H groups) and 3640.5 cm^{-1} (C-O). These vibration behaviors are strongly related with the organic linkers. More importantly, the amalgamation group at 541.9 cm^{-1} and 589.3 cm^{-1} can be observed, which are originated from Co-O and Mn-O symmetric stretching vibration in the octahedral structure (each metal atom is fully coordinated with six oxygen atoms). Importantly, the $Co_{0.8}Mn_{0.2}$-MS displays a similar FT-IR fingerprint before and after OER test, indicating no organic linker structure destruction occurs.

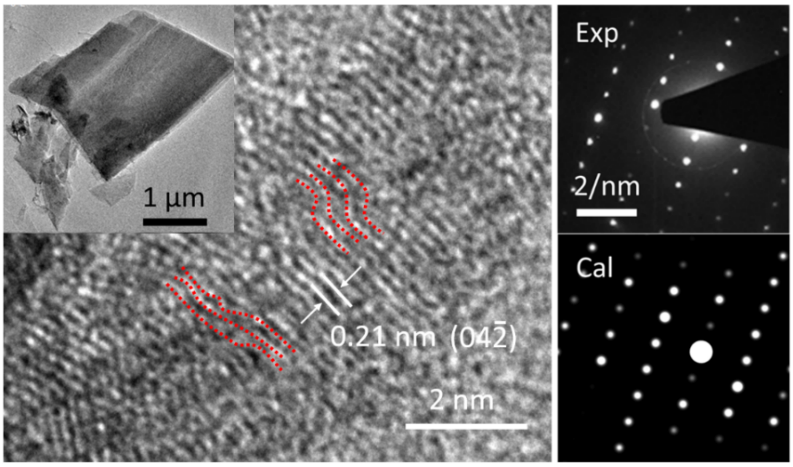

Supplementary Figure 15. The HR-TEM image (left part) and the SAED pattern (right part) of $Co_{0.8}Mn_{0.2}$-MS after OER test. Inset: the corresponding TEM image.

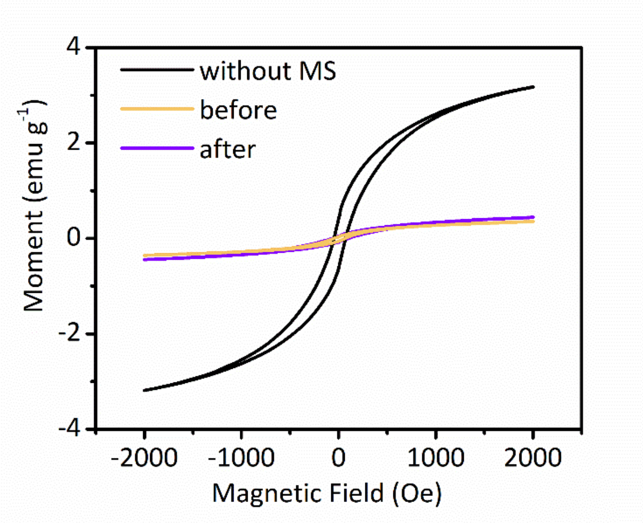

Supplementary Figure 16. Room temperature magnetic hysteresis loops of $Co_{0.8}Mn_{0.2}$-MS 20 min before and after OER testing. Compared to the pristine sample (black lines), the saturation magnetization intensity and coercivity are decreased simultaneously as MS operation, owing to a partial magnetic structure transition from high-spin (HS) state to low-spin (LS) state. However, the magnetic hysteresis loops of $Co_{0.8}Mn_{0.2}$-MS (yellow lines) are coincided basically with that of after 200 h electrocatalytic activity test (pink lines), indicating that the spin reconfiguration is stable.

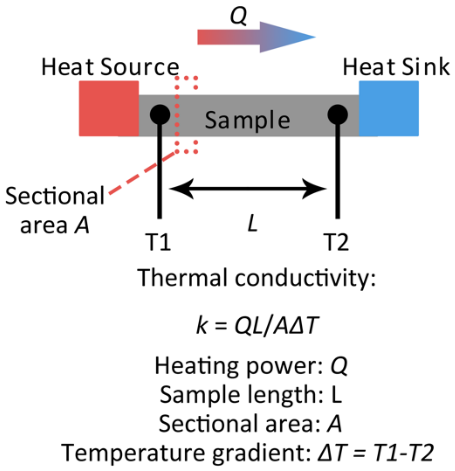

Supplementary Figure 17. Schematic diagram of the device connection in this work.

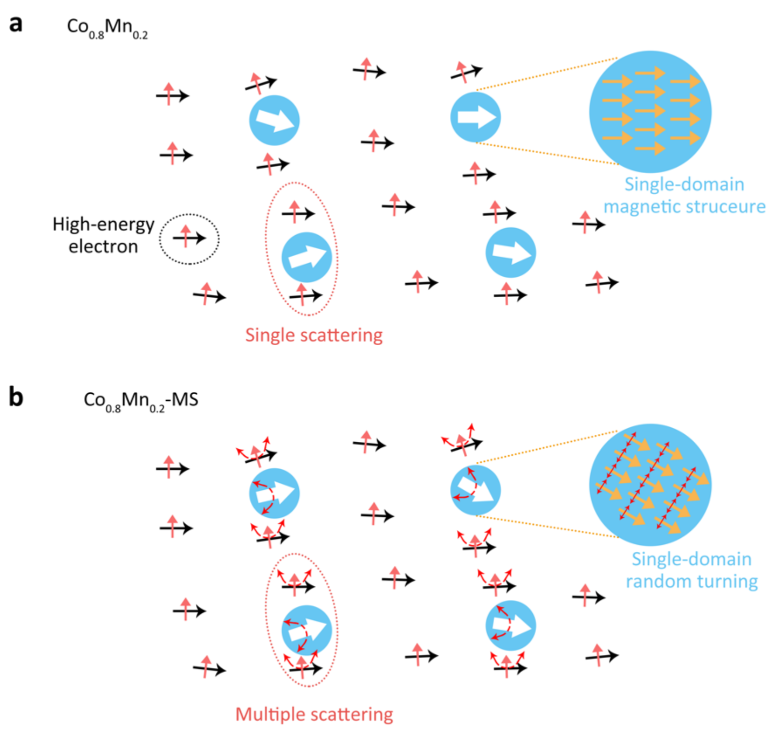

Supplementary Figure 18. (a) Single scattering of electrons as a result of the single-domain spin coupling of the $Co_{0.8}Mn_{0.2}$-MOF, in which the magnetic moment is rigid and not affected by the spin of the high energy conduction carriers. The small black and red arrows are used to illustrate the travel direction of high-energy electron and magnetic domain. (b) Multiple scattering of electrons (indicated by the small red dashed arrows) as a result of the random turning of magnetic domains (indicated by the small red dashed arrows) in the $Co_{0.8}Mn_{0.2}$-MOF with MS.

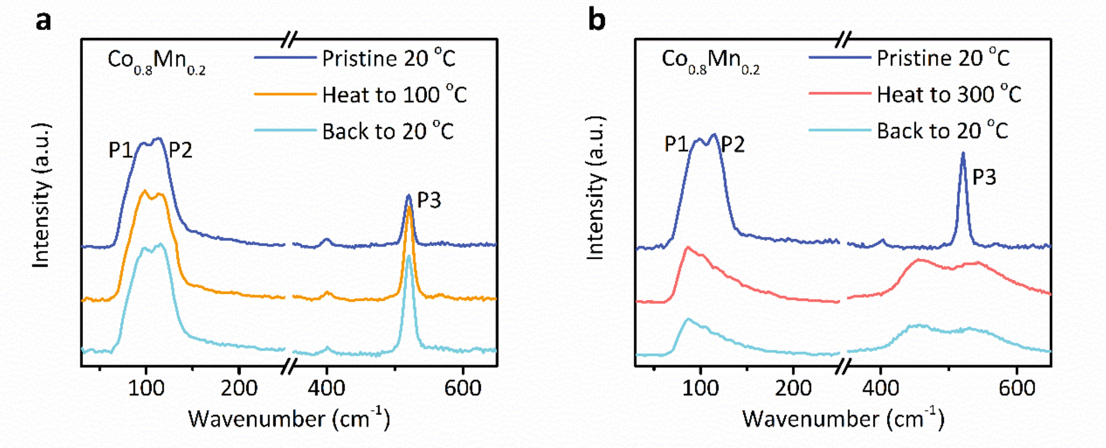

Supplementary Figure 19. The in-situ Raman spectra of $Co_{0.8}Mn_{0.2}$-MOF with different annealing treatment. When the sample is heated to 100 °C by an external heat source, as shown in Supplementary Figure 19(a), the relative intensities of P1 and P2 mode is changed correspondingly, which can be attributed to the lattice thermal strain. After removing the heat source, this Raman fingerprint will recover to its initial status, displaying a reversible behavior. If the ambient temperature is enhanced to 300 °C in Supplementary Figure 19(b), the P3 mode is decomposed into double-peak feature and it cannot back to the original state after withdrawing heat source. This is due to the fact that the crystalline structure has been destroyed, which is consistent with the observation in Supplementary Figure 20.

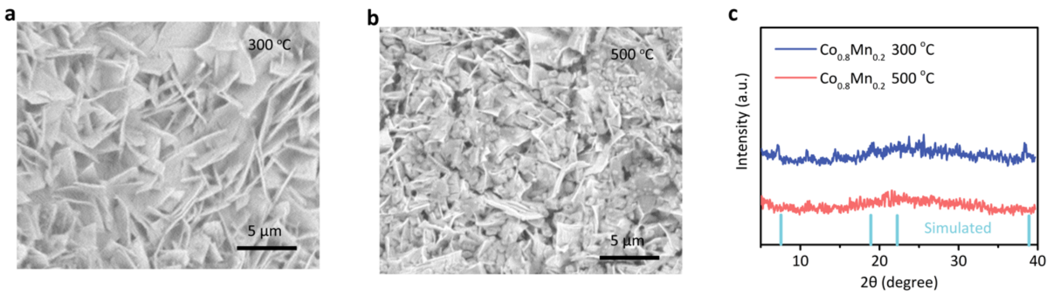

Supplementary Figure 20. FE-SEM images of the $Co_{0.8}Mn_{0.2}$-MOF with the annealing temperature of 300 °C (a) and 500 °C (b). (c) The corresponding XRD spectra of the $Co_{0.8}Mn_{0.2}$-MOF.

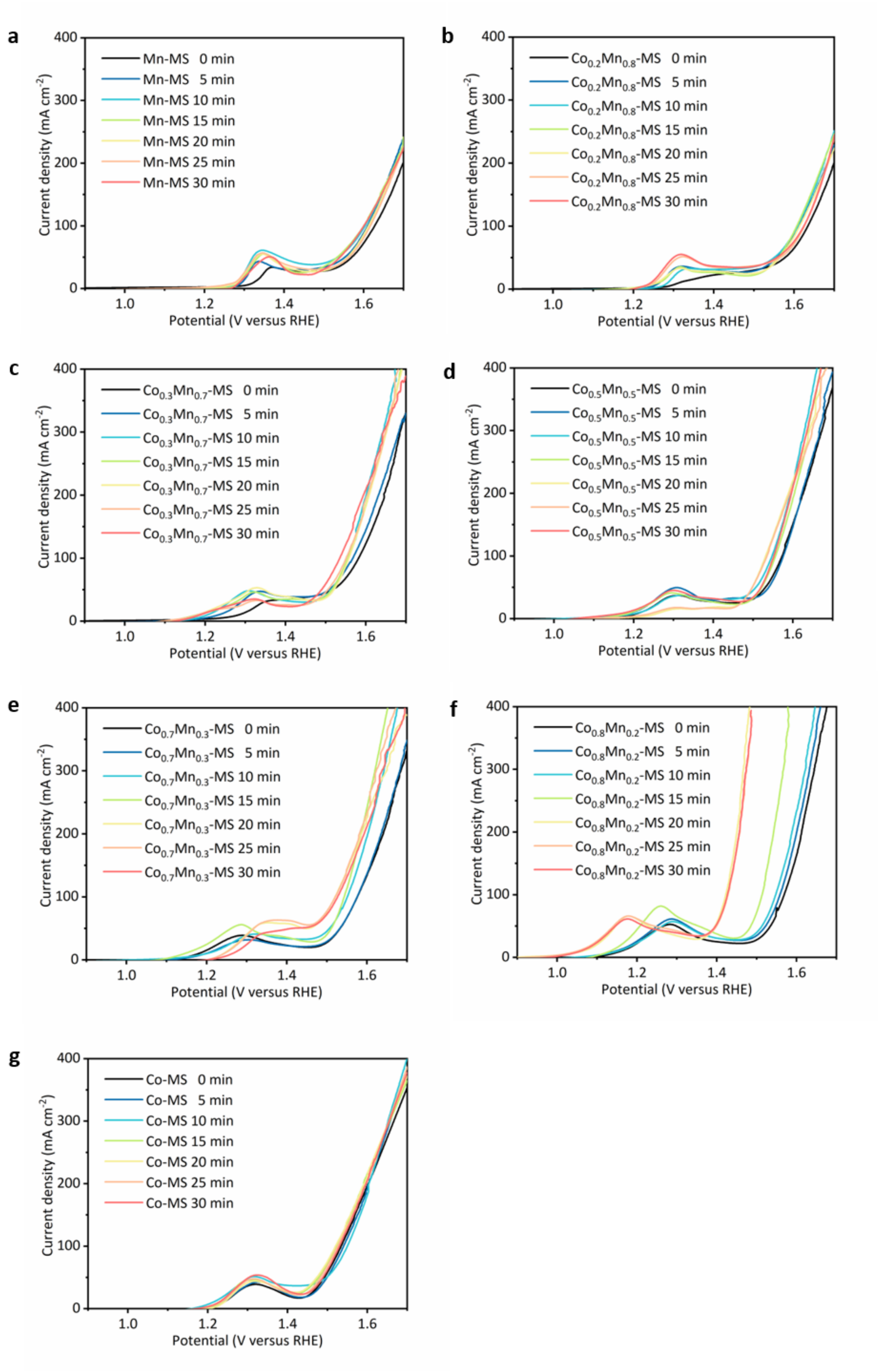

Supplementary Figure 21. Polarization curves of the Co_xMn_y-MOF with different MS time.

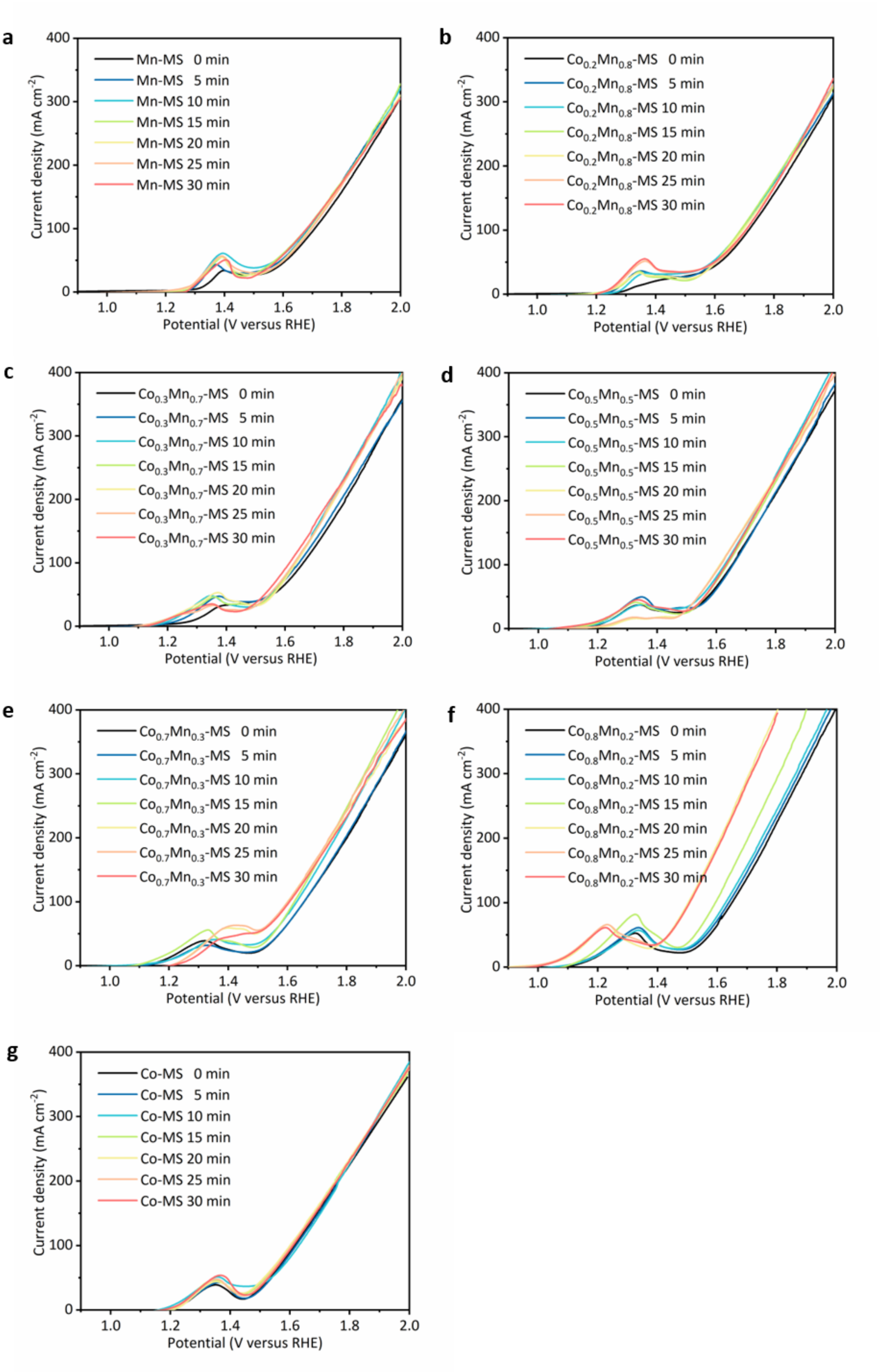

Supplementary Figure 22. The pristine polarization curves without iR compensation.

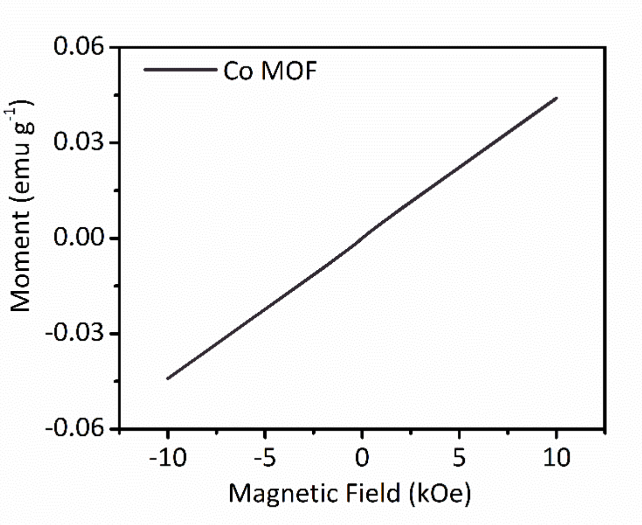

Supplementary Figure 23. M-H curves of the Co-MOF measured at room temperature.

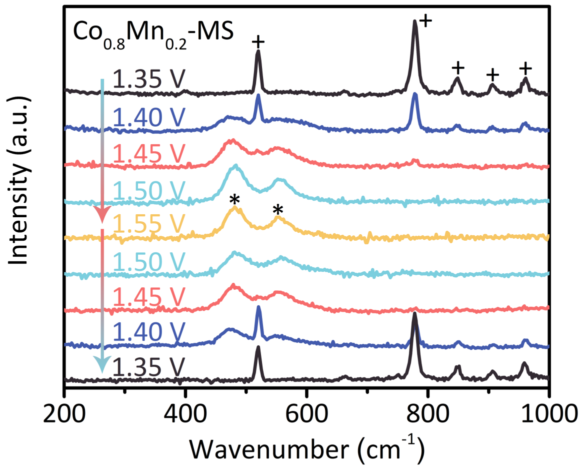

Supplementary Figure 24. Raman spectra of the $Co_{0.8}Mn_{0.2}$-MOF sample with different applied potential. When a potential of 1.4 V is applied, an intermediate (*O) at catalyst surface can be observed by in situ Raman spectra, in which the generated Co-O bonds will lead to two additional broad Raman peaks (marked by *) at 477 and 623 cm^{-1}. When applied potentials are decreased, the Raman signal from this intermediate of Co-O bonds at catalyst surface will disappear. This is because that the lower overpotential cannot start an OER process. Finally, the catalysts will return to the initial state after finishing OER test, thus no relative signal about this intermediate can be observed in the subsequent microstructural characterizations.

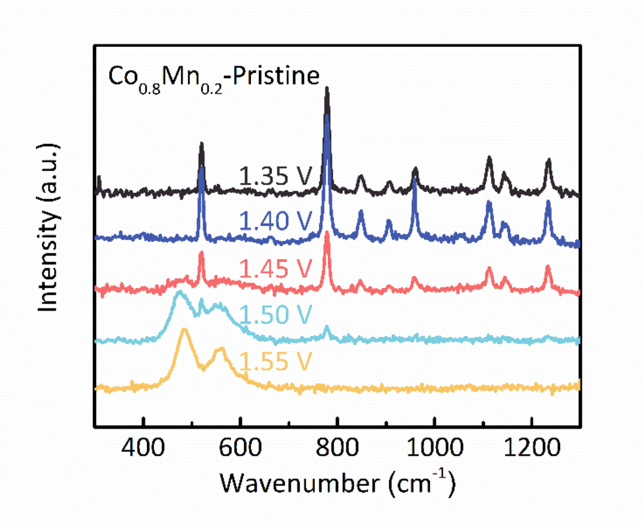

Supplementary Figure 25. Raman spectra of the $Co_{0.8}Mn_{0.2}$-MOF sample with different applied potential.

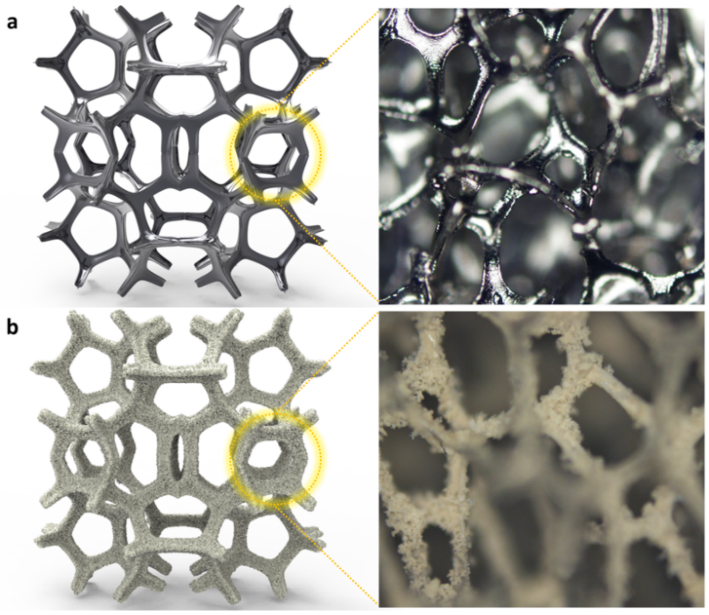

Supplementary Figure 26. Schematic illustration (left) and digital photograph (right) of NF (a) and MOF/NF (b) electrodes. The analysis about schematic illustration and digital photograph of NF (a) and MOF/NF (b) electrodes show that the amount of MOF deposited on NF can be obtained by the quantity difference between NF and MOF/NF. The surface area of NF is about S=1 cm^2. Then the amount of MOF can be calculated to be: $\emptyset = \frac{\mathrm{m(MOF/NF)-m(NF)}}{\mathrm{S}} = 1.8\ mg/cm^2$.

Supplementary Methods

Characterization

The morphology and microstructure were characterized by high-resolution transmission electron microscopy (JEOL2100), field-emission scanning electron microscopy (ZEISS, G500) equipped with X-ray energy dispersive spectrum (EDS), X-ray diffractometer (Bruker, D8) with Cu Kα radiation (λ=0.154 nm) and tapping-mode atomic force microscope (Nanoscope IIIA). In situ Raman spectra were recorded on a Raman microscope (NR-1800, JASCO) using a 514.5 nm argon ion laser with a probe station. XPS measurements were performed on an PHI5000 Versa Probe XPS with the exciting source of Al–Kα and corrected for specimen charging by referencing the C 1s to 284.5 eV. The concentration of metal species was determined by inductively coupled plasma (ICP) atomic emission spectroscopy using Atomscan Advantage (Thermo Jarrell Ash).

Finite element methods for simulation of $Co_{0.8}Mn_{0.2}$-MOFs

Thermal field changes of MOF nanosheet under alternating magnetic field were simulated using the commercial software COMSOL multiphysics. The three-dimensional (3D) geometry of MOF nanosheet was defined according to crystal structure. The thermal effect of the nanosheets was modeled by induction heating multiphysics interface, and the electromagnetic power dissipation of metal elements as a heat source was added into the multiphysics couplings. We used the Maxwell equations to describe the electromagnetic field i.e.:

$$\nabla \times \boldsymbol{H} = \boldsymbol{J} \tag{1}$$

$$\boldsymbol{B} = \nabla \times \boldsymbol{A} \tag{2}$$

$$\boldsymbol{J} = \sigma\boldsymbol{E} + j\omega\boldsymbol{D} + \sigma\boldsymbol{v} \times \boldsymbol{B} + \boldsymbol{J_e} \tag{3}$$

$$\boldsymbol{E} = -j\omega\boldsymbol{A} \tag{4}$$

where ∇ is the Hamiltonian, $\boldsymbol{H}$ is the magnetic field intensity, $\boldsymbol{J}$ is the current density, $\boldsymbol{B}$ is the magnetic flux density, $\boldsymbol{A}$ is the magnetic vector potential, σ is the electrical conductivity, E is the electric field intensity, j is the imaginary number, ω is the angular frequency, $\boldsymbol{D}$ is the electric displacement vector, $\boldsymbol{v}$ is the velocity of the conductor, and $\boldsymbol{J_e}$ is the an externally generated current density. Besides, the heat conduction equation was utilized to calculate the heat transmission and thermal field kinetics as follows:

$$\rho C_p \frac{\partial T}{\partial t} + \rho C_p \mathbf{u} \cdot \nabla T + \nabla \cdot \boldsymbol{q} = Q + Q_{ted} \tag{5}$$

where ρ is the density, C_p is the specific heat capacity, T is temperature, t is the time, **u** is the velocity field (Darcy velocity), $\boldsymbol{q}$ is the conductive heat flux, Q is the heat source, and Q_{ted} is the thermoelastic damping heat source. The $\boldsymbol{q}$ obeys the Fourier's Law:

$$\boldsymbol{q} = -k\nabla T \tag{6}$$

where k is the thermal conductivity. The intensity of the background magnetic field was 0.1 T along the z axis, and the frequency was 150 kHz. Initial temperature was 293 K, and the boundary of MOF nanosheet was set to be thermal insulation. The size of the mesh in the numerical simulation was locally refined to obtain high precision results. Slice of the temperature distribution along yz-plane was shown in Fig. 1e.
